# Multivariable clinical-genetic model for predicting dyskinesia in early-onset Parkinson’s disease

**DOI:** 10.1186/s40035-021-00251-4

**Published:** 2021-07-29

**Authors:** Yong-Ping Chen, Ru-Wei Ou, Xiao-Jing Gu, Ling-Yu Zhang, Bei Cao, Yan-Bing Hou, Kun-Cheng Liu, Jun-Yu Lin, Qian-Qian Wei, Bi Zhao, Ying Wu, Hui-Fang Shang

**Affiliations:** Department of Neurology, Laboratory of Neurodegenerative Disorders, Rare Disease Center, West China Hospital, Sichuan University, Chengdu, 610041 China

## Main text

The levodopa-induced dyskinesias (LIDs) in Parkinson’ disease (PD) patients during levodopa treatment can lead to significant disability. Accumulative evidence has suggested that the younger the age of onset, the more likely the development of LIDs [1]. Till now, most of the studies on clinical or genetic risk factors for LIDs were cross-sectional [2], or included limited sample size [3], or mainly included late-onset PD patients [4]. Here, we investigated the incidence of LIDs in the early stage of early-onset PD (EOPD), including the first 5 years of duration and the first 5 years of dopamine replacement therapy (DRT), and established and validated clinical-genetic models for LID prediction. Detailed methods are provided in Supplementary File 1.

A total of 279 EOPD patients with age at onset < 50 years were included as the derivation cohort (Table S1), and 99 of them developed LIDs during follow-up at the time of analysis, including peak-dose dyskinesias (68.7%), diphasic dyskinesias (7.1%) and “off”-state dystonia (27.3%). The detailed flowchart of this study, including the derivation cohort and the validation cohort, is shown in Fig. S1. Eleven single nucleotide polymorphisms (SNPs), as the candidate genetic predictors, are shown in Table S2. The levodopa equivalent daily dose (LEDD) was significantly different between LID and non-LID groups at baseline (*P* < 0.001). A total of 43 EOPD patients developed LIDs (15.4%) in the first 5 years of duration, and 56 patients developed LIDs after over 5 years of disease duration; and the former patients had a shorter duration at baseline than the latter ones (Table S3). In addition, of the 232 patients who received DRT for more than 5 years, 69 patients (29.7%, 69/232) developed LIDs in the first 5 years of DRT. Both the incidence of LIDs at the first 5 years of duration and at the first 5 years of DRT in the derivation cohort were similar with those in the validation cohort (*n* = 144) (16.0% and 30.4%, respectively, Table S4).

**Fig. 1:**
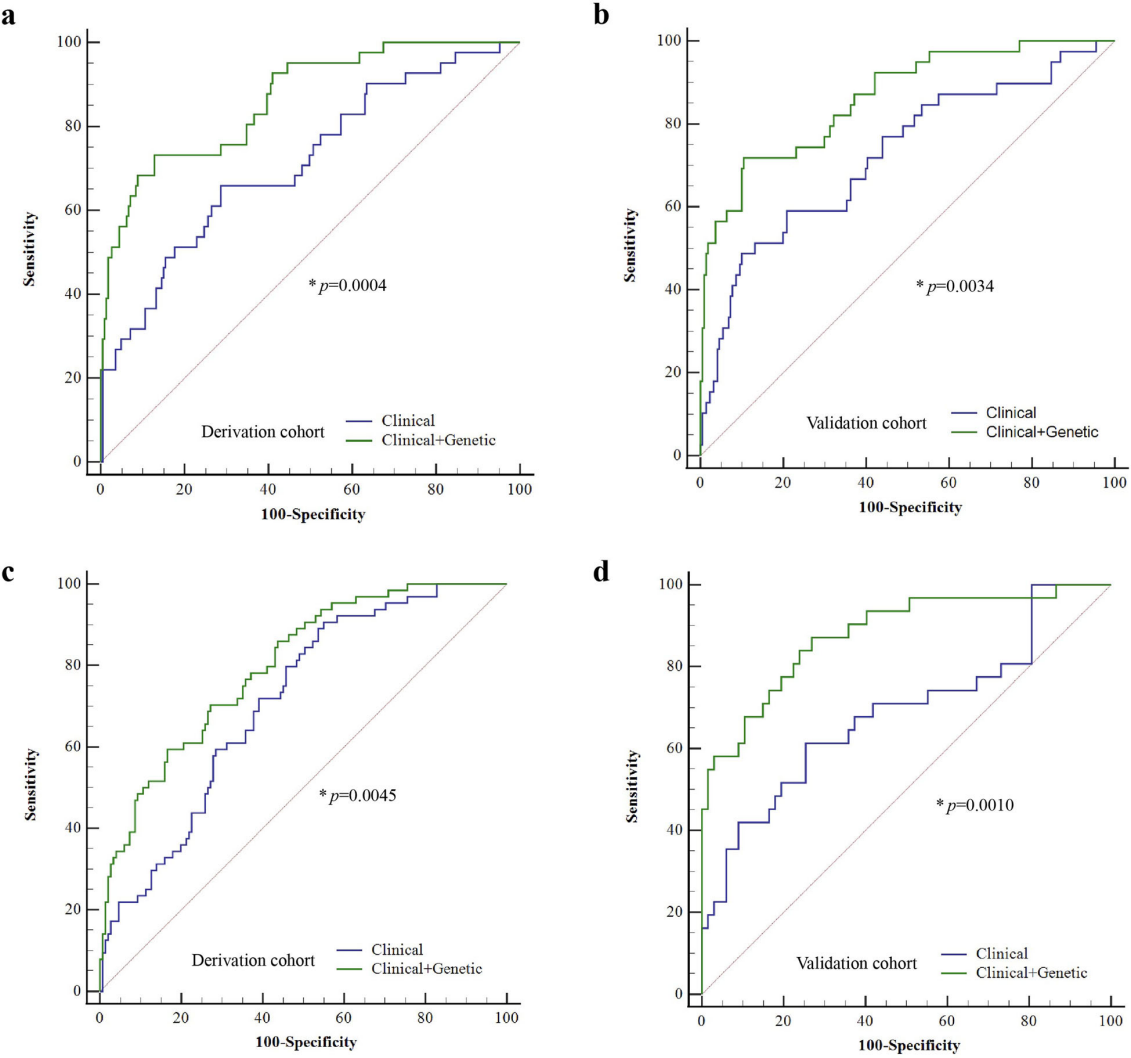
ROC curves for prediction of LID incidence during the first 5 years of duration (**a**, **b**) and during the first 5 years of DRT (**c**, **d**), using two different models in the derivation (**a**, **c**) and the validation groups (**b**, **d**). Clinical variables included sex, onset age, duration, initial treatment, LEDD, initial symptoms, BMI, UPDRS-III, H&Y staging, hyposmia, and family history. Genetic variables included genotype data on 11 candidate SNPs. **a** Clinical model AUC = 0.714, 95%CI, 0.655–0.767; clinical-genetics model AUC = 0.864, 95%CI, 0.817–0.903; *n* = 279. **b** Clinical model AUC = 0.740, 95%CI, 0.657–0.811; clinical-genetics model AUC = 0.884, 95%CI, 0.817–0.932; *n* = 144. **c** Clinical model AUC = 0.710, 95%CI, 0.645–0.770; clinical-genetics model AUC = 0.798, 95%CI, 0.738–0.850; *n* = 232. **d** Clinical model AUC = 0.687, 95%CI, 0.585–0.777; clinical-genetics model AUC = 0.879, 95%CI, 0.798–0.936; *n* = 102

Receiver operating characteristic curves plotted using 11 clinical variables with or without genetic variables were analyzed. For LIDs at the first 5 years of duration, the area under the curve (AUC) was 0.71 (95%CI: 0.66–0.77) when only clinical variables were used, and increased to 0.86 (95%CI: 0.82–0.90) (*P* = 0.0004) when genotype data of 11 candidate SNPs were added (Fig. 1a). The established prediction models were confirmed in the validation group, with AUC of 0.74 (95%CI: 0.66–0.81) when only clinical variables were used, which increased to 0.88 (95%CI: 0.82–0.93) (*P* = 0.0034) when genotype data were added (Fig. 1b). There was no significant difference in AUC in the clinical model or clinical-genetic model between the derivation and the validation groups (Table S5). For LIDs occurring at the first 5 years of DRT, the AUC was 0.71 (95%CI: 0.65–0.77) when only clinical variables were used, and increased to 0.80 (95%CI: 0.74–0.85) (*P* = 0.0045) when genotype data were added (Fig. 1c). In the validation group, the AUC was 0.69 (95%CI: 0.59 to 0.78) when only clinical variables were used, and increased to 0.88 (95%CI: 0.80–0.94) (*P* = 0.0010) when genotype data were added (Fig. 1d). There was no significant difference in AUC in the clinical model or the clinical-genetic model between the derivation and the validation groups (Table S5). In addition, there were no significant differences in AUCs for LID incidence during the first 5 years of duration or during the first 5 years of DRT, based on either model, between the derivation group and the subgroups excluding patients with PD-causative gene mutations (Table S6).

Backward stepwise regression showed that for LID incidence at the first 5 years of duration, the clinical predictors included LEDD and disease duration, and the genetic predictors included *DRD2*, *DRD3*, *SLC6A3*, *HRAS, COMT* and *BDNF* genotypes. Further multivariate logistic regression analysis showed that LEDD at baseline was significantly associated with LID incidence (*P* = 3.4e-7). The minor homozygous genotype of *DRD3* rs6280 increased LID incidence (*P* = 0.001, OR = 7.2), while the heterozygous genotype of *HRAS* rs12628 significantly decreased LID incidence (*P* = 0.005, OR = 0.22) (Table S7). For LID incidence during the 5 years of DRT, LEDD significantly increased the risk of LIDs (*P* = 1.7e-4).

In our cohort, the incidence of LIDs was 15.4% in the first 5 years of duration, and increased to 29.7% in the first 5 years of DRT. In a previous study, the incidence of LIDs has been reported to be 21.7% in Chinese LOPD patients [5], suggesting that the LIDs are common in EOPD, and EOPD patients are susceptible to the development of LIDs. Importantly, the AUCs in the clinical or clinical-genetic model for predicting LID incidence remained consistent after excluding patients carrying PD causative gene variants, suggesting that the established clinical-genetic model is potentially useful for prediction of LIDs in all EOPD patients. In addition, the multivariate logistic regression results on LEDD are consistent with the recent finding that LEDD is the most strong clinical variable for predicting LIDs [4] and with the recommendation of a low initial dosage of LEDD for PD in China [6]. The *DRD3* rs6280 GG genotype, as the strongest genetic variable, increased the risk of LIDs compared with the AA genotype in our cohort, which was consistent with the finding from a Korean cohort [7]. The distinct responses to levodopa may be attributed to the lower affinity of the AA genotype to dopamine [8].

In conclusion, the established clinical-genetic models and the identified independent predictors in the early stage of EOPD in Chinese patients may have good application prospects for LID prediction and DRT management.

## Supplementary Information

**Additional file 1.**

**Additional file 2: Table S1.** Baseline characteristics of patients in the derivation cohort. **Table S2. **Characteristics of the selected genetic variants. **Table S3.** Baseline characteristics of patients with LIDs in the derivation cohort. **Table S4.** Baseline characteristics of patients in the validation cohort. **Table S5.** Comparison of different prediction models between the derivation group and the validation group. **Table S6.** Comparison of different prediction models between the derivation group and subgroups excluding PD-causative gene mutations. **Table S7.** Single-factor association with LID incidence in the multivariable non-linear models. **Fig. S1.** Flowchart of the study.

## Data Availability

The datasets used and/or analyzed during the current study are available from the corresponding author on reasonable request.

## Abbreviations

PD: Parkinson’s disease
EOPD: Early-onset PD
LOPD: Late-onset PD
LIDs: Levodopa-induced dyskinesias
DRT: Dopamine replacement therapy
