# Supplementary material for: Multivariable clinical-genetic model for predicting dyskinesia in early-onset Parkinson’s disease
Source: Transl Neurodegener. 2021 Jul 29;10:26. doi: 10.1186/s40035-021-00251-4 (PMC8320054; doi:10.1186/s40035-021-00251-4)
Supplement: Supplementary file 1 — Additional file 1. [file 40035_2021_251_MOESM1_ESM.docx]

**Supplemental file 1**

**Materials and Methods**

***Subjects***

In the derivation group, a total of 304 early-onset Parkinson’s disease (EOPD) patients (age of onset ≤ 50 years) who admitted to the Department of Neurology, West China Hospital, between Dec. 2010 and Dec. 2018 participated in the prospective, longitudinal cohort study to investigate the incidence, clinical and genetic characteristics and prognosis of EOPD. All the subjects provided informed consent prior to participating and the study was approved by the ethics committee of West China Hospital, Sichuan University. In order to investigate the incidence of LIDs in the early stage of EOPD, the disease duration of participants with EOPD was required to be < 5 years and participants were excluded if they already presented with LIDs at baseline. Diagnostic procedures for PD were based on the UK Brain Bank Clinical Diagnostic Criteria (1) or 2015 Movement Disorder Society (MDS) Clinical Diagnostic Criteria (2). All the participants were followed up at least once by professional neurologists through a face-to-face. Participants were excluded if they presented with atypical Parkinson disease or other secondary parkinsonian syndromes during the follow-up visits. The main endpoint of follow-up in the current study was the occurrence of dyskinesia according to a previous study (3), including peak‐dose dyskinesias (PDD), diphasic dyskinesias (DD), and “Off” state dystonia (OSD). Demographic and clinical data were collected as described in our previously study (4). At the end of the study, 25 participants who were diagnosed with atypical Parkinson disease during follow-up or were lost to follow-up were excluded from the current study. Finally, a total of 279 patients who received DRT at recruitment or during follow-up, and had complete data were performed statistical analyses and genetic evaluation (**Fig. S1**). In addition, another cohort including 144 EOPD from our center who met the mentioned criterion was used as the validation group (**Fig. S1**).

***Candidate genes and variants selection***

Genomic DNA was collected from peripheral blood leukocytes using standard phenol-chloroform procedures. As described above, dysregulation of dopamine metabolism might be one of the main reasons of LID incident (5), in the clinical-genetic model analysis, our selected candidate genes involved in monoaminergic system, dystonia or LIDs incident reported in the published literature, including 1) four genes of dopamine receptor or dopamine transporter, *DRD1, DRD2*, *DRD3* and *SLC6A3*; 2) one dopamine receptor expression regulation gene, *BDNF*; 3) two dopamine metabolism genes, *TH* and *COMT*; 4) two other receptors genes, *OPRM1*, *GRIN2B*; 5) one channel relative gene, *HRAS*; 6) one DYT gene, *TOR1A*. For each gene, all candidate variants were obtained from the Infinium Asian Screening Array (IASA) as our previous study(6). Variants selected have to meet the predefined criteria: 1) variants or genes have been associated with LIDs, or dopamine receptor, or dopamine metabolism or dystonia as reported in literatures; 2) The minor allele frequency (MAF) of each variant is more than 0.05; 3) functional category was preferential considerations for each step when we filtered the variants, as following: missense > coding synonymous > 3’-UTR/5’-UTR > Intron. Finally, eleven single nucleotide polymorphisms (SNPs), including seven reported associated with LIDs in PD (*DRD1* rs4532 (7), *DRD2* rs1800497 (8), *DRD3* rs6280 (9), *BDNF* rs6265 (10), *COMT* rs4680 (11), *OPRM1* rs1799971(12) and *HRAS* rs12628 (13)), *SLC6A3* rs460000 (which is linkage equilibrium with rs28363170 which was reported to be associated with dyskinesia in PD (14)), *TOR1A* rs1801968 (which was reported to be associated with dystonia (15)), *TH* rs6356 and *GRIN2B* rs1806201, were selected (**Table S2**).

In addition, genotype-phenotype analysis in previous studies suggested some PD causative genes were related to LIDs incident for PD, such as *Parkin* (16), *PINK1* and *DJ1* (17), we also re-analyzed and compared clinical or clinical-genetic models of LIDs incident when patients with PD causative genes mutations (including *SNCA*, *Parkin*, *PINK1*, *DJ1*, *LRRK2*, *PLA2G6* and *VPS35*, all of which were suggested to be as the most high-confidence Mendelian PD genes(18)) were excluded or not. As our previous study (6), the multiplex ligation-dependent probe amplification (MLPA) and whole exome sequencing (WES) were performed to detected rare variants in the coding regions of all the seven genes and the exon deletions/insertions of *SNCA*, *PARK2*, *PINK1*, *DJ1 and LRRK2* for patients from the derivation group.

***Statistical analysis***

Incident LIDs predictability was estimated with receiver operating characteristic (ROC) curve, eleven clinical variables, some of which were reported to be associated with LIDs incident in PD (19, 20), including sex, age of onset, duration, initial treatments (LDA or other DRT), LEDD, initial symptoms, body mass indices (BMI), Unified Parkinson Disease Rating Scale - III (UPDRS-III) score, Hoehn-Yahr (H&Y) stage score, hyposmia, and family history were included. To assess whether inclusion of genotype information would improve predictability of LIDs incident, ROC curves were plotted with the preselected clinical variables only, and then with candidate SNPs added. Area under the curves (AUCs) were compared using Delong’s test for two correlated ROC curves (between clinical and clinical-genetic models) from the same set of patients. The comparisons for AUCs of ROC among clinical or clinical-genetic models from independent groups of patients were performed by MedCalc software (<https://www.medcalc.org/manual/independentROCcurves.php>) (21). Adjusted single factors predicting LIDs incident were assessed using logistic regression models, with variables selection carried out in a backward-stepwise procedure. That is to say, ROC curves analysis included all the preselected clinical variables and all candidate SNPs, but for independent factor associations analysis, we only included the variables (clinical variables and candidate SNPs) whose p value less than 0.15 from logistic regression of ROC curves analysis.

***Statistical Analysis***

The comparison of continuous data was assessed using Student’s t-test. Chi-square tests were used to compare categorical variables. A two-tailed p-value < 0.05 was considered statistically significant. Statistical analysis was performed using SPSS version 25.0 (SPSS, Chicago, IL, USA). A Bonferroni correction for multiple comparisons was performed if appropriate.

***Data availability***

Qualified researchers may obtain de-identified data used for this study from corresponding author and the study team upon reasonable request and West China Hospital Institutional Review Board approval.

**References:**

1. Hughes AJ, Daniel SE, Kilford L, Lees AJ. Accuracy of clinical diagnosis of idiopathic Parkinson's disease: a clinico-pathological study of 100 cases. J Neurol Neurosurg Psychiatry. 1992;55(3):181-4.

2. Postuma RB, Berg D, Stern M, Poewe W, Olanow CW, Oertel W, et al. MDS clinical diagnostic criteria for Parkinson's disease. Mov Disord. 2015;30(12):1591-601.

3. Luquin MR, Scipioni O, Vaamonde J, Gershanik O, Obeso JA. Levodopa-induced dyskinesias in Parkinson's disease: clinical and pharmacological classification. Mov Disord. 1992;7(2):117-24.

4. Liu K, Ou R, Hou Y, Wei Q, Cao B, Song W, et al. Predictors of Pisa syndrome in Chinese patients with Parkinson's disease: A prospective study. Parkinsonism Relat Disord. 2019;69:1-6.

5. Calabresi P, Di Filippo M, Ghiglieri V, Tambasco N, Picconi B. Levodopa-induced dyskinesias in patients with Parkinson's disease: filling the bench-to-bedside gap. Lancet Neurol. 2010;9(11):1106-17.

6. Chen Y, Gu X, Ou R, Zhang L, Hou Y, Liu K, et al. Evaluating the Role of SNCA, LRRK2, and GBA in Chinese Patients With Early-Onset Parkinson's Disease. Mov Disord. 2020.

7. Dos Santos EUD, Duarte EBC, Miranda LMR, Asano AGC, Asano NMJ, Maia MMD, et al. Influence of DRD1 and DRD3 Polymorphisms in the Occurrence of Motor Effects in Patients with Sporadic Parkinson's Disease. Neuromolecular Med. 2019;21(3):295-302.

8. Wang J, Liu ZL, Chen B. Association study of dopamine D2, D3 receptor gene polymorphisms with motor fluctuations in PD. Neurology. 2001;56(12):1757-9.

9. Lee JY, Cho J, Lee EK, Park SS, Jeon BS. Differential genetic susceptibility in diphasic and peak-dose dyskinesias in Parkinson's disease. Mov Disord. 2011;26(1):73-9.

10. Foltynie T, Cheeran B, Williams-Gray CH, Edwards MJ, Schneider SA, Weinberger D, et al. BDNF val66met influences time to onset of levodopa induced dyskinesia in Parkinson's disease. J Neurol Neurosurg Psychiatry. 2009;80(2):141-4.

11. Sampaio TF, Dos Santos EUD, de Lima GDC, Dos Anjos RSG, da Silva RC, Asano AGC, et al. MAO-B and COMT Genetic Variations Associated With Levodopa Treatment Response in Patients With Parkinson's Disease. J Clin Pharmacol. 2018;58(7):920-6.

12. Strong JA, Dalvi A, Revilla FJ, Sahay A, Samaha FJ, Welge JA, et al. Genotype and smoking history affect risk of levodopa-induced dyskinesias in Parkinson's disease. Mov Disord. 2006;21(5):654-9.

13. Martin-Flores N, Fernandez-Santiago R, Antonelli F, Cerquera C, Moreno V, Marti MJ, et al. MTOR Pathway-Based Discovery of Genetic Susceptibility to L-DOPA-Induced Dyskinesia in Parkinson's Disease Patients. Mol Neurobiol. 2019;56(3):2092-100.

14. Kaiser R, Hofer A, Grapengiesser A, Gasser T, Kupsch A, Roots I, et al. L -dopa-induced adverse effects in PD and dopamine transporter gene polymorphism. Neurology. 2003;60(11):1750-5.

15. Chen Y, Burgunder JM, Song W, Huang R, Shang HF. Assessment of D216H DYT1 polymorphism in a Chinese primary dystonia patient cohort. Eur J Neurol. 2012;19(6):924-6.

16. Wickremaratchi MM, Knipe MD, Sastry BS, Morgan E, Jones A, Salmon R, et al. The motor phenotype of Parkinson's disease in relation to age at onset. Mov Disord. 2011;26(3):457-63.

17. Kasten M, Hartmann C, Hampf J, Schaake S, Westenberger A, Vollstedt EJ, et al. Genotype-Phenotype Relations for the Parkinson's Disease Genes Parkin, PINK1, DJ1: MDSGene Systematic Review. Mov Disord. 2018;33(5):730-41.

18. Blauwendraat C, Nalls MA, Singleton AB. The genetic architecture of Parkinson's disease. Lancet Neurol. 2019.

19. Tran TN, Vo TNN, Frei K, Truong DD. Levodopa-induced dyskinesia: clinical features, incidence, and risk factors. J Neural Transm (Vienna). 2018;125(8):1109-17.

20. Warren Olanow C, Kieburtz K, Rascol O, Poewe W, Schapira AH, Emre M, et al. Factors predictive of the development of Levodopa-induced dyskinesia and wearing-off in Parkinson's disease. Mov Disord. 2013;28(8):1064-71.

21. Hanley JA, McNeil BJ. The meaning and use of the area under a receiver operating characteristic (ROC) curve. Radiology. 1982;143(1):29-36.

22. Bender R, Lange S. Adjusting for multiple testing--when and how? J Clin Epidemiol. 2001;54(4):343-9.
