# Supplementary material for: Multivariable clinical-genetic model for predicting dyskinesia in early-onset Parkinson’s disease
Source: Transl Neurodegener. 2021 Jul 29;10:26. doi: 10.1186/s40035-021-00251-4 (PMC8320054; doi:10.1186/s40035-021-00251-4)
Supplement: Supplementary file 2 — Additional file 2: Table S1. Baseline characteristics of patients in the derivation cohort. Table S2. Characteristics of the selected genetic variants. Table S3. Baseline characteristics of patients with LIDs in the derivation cohort. Table S4. Baseline characteristics of patients in the validation cohort. Table S5. Comparison of different prediction models between the derivation group and the validation group. Table S6. Comparison of different prediction models between the derivation group and subgroups excluding PD-causative gene mutations. Table S7. Single-factor association with LID incidence in the multivariable non-linear models. Fig. S1. Flowchart of the study. [file 40035_2021_251_MOESM2_ESM.docx]

**Supplemental file 2**

**Table S1 Baseline characteristics of patients analyzed in the derivation cohort**

| Variable | Total | LIDs | Non-LIDs | *P*-value^#^ |
| --- | --- | --- | --- | --- |
| N | 279 | 99 | 180 | - |
| Sex, % male (male: female) | 54.5% (152:127) | 45.5% (45:54) | 59.1% (107:73) | 0.025 |
| Age, years | 44.5±6.2 | 43.7±6.3 | 45.0±6.1 | 0.095 |
| BMI | 22.9±3.2 | 23.0±3.3 | 22.8±3.1 | 0.688 |
| Family history, % (Y: N) | 12.9% (36: 243) | 9.1% (9:89) | 14.9% (27:154) | 0.173 |
| Onset age, years | 42.0±6.2 | 41.3±6.7 | 42.4±5.9 | 0.161 |
| Duration of PD at baseline, years | 2.3±1.7 | 2.3±1.8 | 2.4±1.8 | 0.443 |
| Initial symptoms (T, R, O) | 125:131:22 | 35:56:8 | 90:75:14 | 0.048 |
| Initial treatment (LDT: non-LDT) | 169: 110 | 69:30 | 100:80 | 0.021 |
| Initial LEDD | 349.4±246.8 | 441.5±214.5 | 298.7±249.3 | <0.001 |
| UPDRS-III | 27.1±16.2 | 27.6±16.2 | 26.8±16.2 | 0.701 |
| H&Y stage | 2.1±0.7 | 2.1±0.7 | 2.1±0.7 | 0.536 |
| Hyposmia, % (Y: N) | 23.7% (66:213) | 23.3% (23:76) | 23.8% (43:137) | 0.902 |
| Duration of follow up | 9.2±4.1 | 8.9±5.0 | 9.3±3.5 | 0.464 |
| Subtype of LIDs (PDD: DD: OSD) | - | 68:7:24 | - | - |
| Retention rate | 91.8% (279/304) | - | - | - |
| *Patients with last follow up visit to disease onset ≥ 5 years, % (*n*/total) | 93.9% (262/279) | - | - | - |
| *Patients receiving DA ≥ 5 years, % (*n*/total) | 77.1% (215/279) | - | - | - |
| Patients with LIDs in the first 5 years of duration | 15.4% (43/279) | - | - | - |
| *Patients with LIDs in the first 5 years after receiving DRT | 29.7% (69/232) | - | - | - |

BMI, body mass indices; T or TD, tremor; R, rigidity; O, bradykinesia, gait disturbance and non-motor symptoms; LDT, levodopa treatment; non-LDT, no levodopa treatment; LEDD, levodopa equivalent daily dose; UPDRS, Unified Parkinson Disease Rating Scale; PDD, Peak‐dose dyskinesias; DD, Diphasic dyskinesias; OSD, “Off” state dystonia; DRT, dopamine replacement therapy; *the remaining 17 patients didn’t continue to been followed up because they presented dyskinesia at the duration of the last follow up visit to disease onset < 5 years. ^#^ Comparison between patients presented LIDs and patients without LIDs.

**Table S2 Characteristics of the selected genetic variants**

| Gene | Function | Variant | Alleles | Position^#^ | Location in gene | MAF* | MAF^&^ |
| --- | --- | --- | --- | --- | --- | --- | --- |
| *DRD3* | dopamine receptor | rs6280 | G/A | chr3:113890815 | Exon, missense | A=0.695 | A=0.665 |
| *DRD1* | dopamine receptor | rs4532 | G/A | chr5:174870150 | 5’ UTR | A=0.859 | A=0.786 |
| *DRD2* | dopamine receptor | rs1800497 | G/A | chr11:113270828 | Exon, missense | A=0.398 | A=0.378 |
| *SLC6A3* | dopamine transporter | rs460000 | G/T | chr5:1432825 | Intron | T=0.518 | T=0.579 |
| *OPRM1* | receptor | rs1799971 | A/G | chr6:154360797 | Exon, missense | G=0.370 | G=0.342 |
| *GRIN2B* | receptor | rs1806201 | C/T | chr12:13717508 | Exon, synonymous | T=0.521 | T=0.507 |
| *HRAS* | channel relative | rs12628 | T/C | chr11:534242 | 5’ UTR | C=0.189 | C=0.205 |
| *TH* | dopamine metabolism | rs6356 | C/T | chr11:2190951 | Exon, missense | T=0.780 | T=0.828 |
| *COMT* | dopamine metabolism | rs4680 | G/A | chr22:19951271 | Exon, missense | A=0.271 | A=0.263 |
| *BDNF* | receptor regulation | rs6265 | C/T | chr11:27679916 | Exon, missense | T=0.491 | T=0.502 |
| *TOR1A* | dystonia-relative | rs1801968 | C/G | chr9:132580901 | Exon, missense | G=0.082 | G=0.099 |

^#^ Position on Genome Reference Consortium human genome build 37 (GRCh37); * MAF, minor allele frequencies from east Asian cohort of GnomAD database; ^&^ MAF from EOPD patients

**Table S3 Baseline characteristics of patients with LIDs in the derivation cohort**

| Variable | LID+(<5 years) | LID+(>5 years) | *P*-value |
| --- | --- | --- | --- |
| N | 43 | 56 | - |
| Sex, % male (male: female) | 48.8% (21:22) | 42.9% (24:32) | 0.554 |
| Age, years | 43.5±6.2 | 43.8±6.4 | 0.828 |
| BMI | 23.5±3.7 | 22.7±3.1 | 0.253 |
| Family history, % (Y: N) | 9.3% (4:39) | 8.9% (5:51) | 1.000 |
| Onset age, years | 41.8±6.0 | 41.0±7.2 | 0.542 |
| Duration of PD at baseline, years | 1.7±1.6 | 2.9±2.6 | 0.016 |
| Initial symptoms (T, R, O) | 14:25:4 | 21:31:4 | 0.561 |
| Initial treatment (LDT: non-LDT) | 30:13 | 39:17 | 0.989 |
| Initial LEDD | 474.0±219.9 | 416.6±208.7 | 0.188 |
| UPDRS-III | 26.4±16.6 | 28.6±15.9 | 0.525 |
| H&Y stage | 2.1±0.7 | 2.1±0.7 | 0.904 |
| Hyposmia, % (Y: N) | 23.3% (10:33) | 23.2% (13:43) | 0.996 |
| Duration of follow up | 6.9±5.0 | 10.5±4.3 | <0.001 |

T, tremor; R, rigidity; O, bradykinesia, gait disturbance and non-motor symptoms; LDT, levodopa treatment; non-LDT, no levodopa treatment; LEDD, levodopa equivalent daily dose; UPDRS, Unified Parkinson Disease Rating Scale; BMI, body mass indices

**Table S4 Baseline characteristics of patients in the validation cohort**

| Variable | Total | LIDs | Non-LIDs | *P*-value |
| --- | --- | --- | --- | --- |
| N | 144 | 45 | 99 | - |
| Sex, % male (male: female) | 41.0% (59:85) | 53.3% (24:21) | 35.4% (35:64) | 0.042 |
| Age, years | 45.7±6.4 | 45.6±5.9 | 45.8±6.6 | 0.923 |
| BMI | 22.6±3.0 | 22.1±2.9 | 22.8±3.1 | 0.173 |
| Family history, % (Y: N) | 27.1% (39: 105) | 20.0% (9:36) | 30.3% (30:69) | 0.064 |
| Onset age, years | 42.4±6.3 | 43.1±5.6 | 42.1±6.6 | 0.395 |
| Duration of PD at baseline, years | 2.8±1.9 | 2.6±2.1 | 2.9±1.7 | 0.237 |
| Initial symptoms (T, R, O) | 75:46:22 | 23:21:8 | 52:32:14 | 0.568 |
| Initial treatment (LDT: non-LDT) | 77: 67 | 32:13 | 45:54 | 0.004 |
| Initial LEDD | 409.5±297 | 508.8±376.0 | 364.4±242.2 | 0.021 |
| UPDRS-III | 25.0±17.1 | 27.0±19.3 | 24.1±16.0 | 0.357 |
| H&Y stage | 1.9±0.7 | 2.0±0.8 | 1.9±0.6 | 0.150 |
| Hyposmia, % (Y: N) | 40.3% (58:86) | 44.4% (20:25) | 38.4% (38:61) | 0.492 |
| Duration of follow up | 8.0±4.5 | 8.1±5.2 | 8.0±4.2 | 0.898 |
| Patients with last follow up visit to disease onset ≥ 5 years, % (*n*/total)* | 92.4% (133/144) |  |  |  |
| Patients with LIDs in the first 5 years of duration | 16.0% (23/144) |  |  |  |
| Patients with LIDs in the first 5 years after receiving DRT | 30.4% (31/102) |  |  |  |

BMI, body mass indices; T or TD, tremor; R, rigidity; O, bradykinesia, gait disturbance and non-motor symptoms; LDT, levodopa treatment; non-LDT, no levodopa treatment; LEDD, levodopa equivalent daily dose; UPDRS, Unified Parkinson Disease Rating Scale; DRT, dopamine replacement therapy; *the remaining 11 patients didn’t continue to been followed up because they presented dyskinesia at the duration of the last follow up visit to disease onset < 5 years.

**Table S5 Comparison of different prediction models between the derivation group and the validation group**

| Models | Derivation group | | |  | Validation group | | |  | *P*-value |
| --- | --- | --- | --- | --- | --- | --- | --- | --- | --- |
|  | AUC | SE | 95%CI |  | AUC | SE | 95%CI |  |  |
| Duration of first 5 years |  |  |  |  |  |  |  |  |  |
| Clinical | 0.714 | 0.0461 | 0.655-0.767 |  | 0.740 | 0.0563 | 0.657-0.811 |  | 0.7209 |
| Clinical-Genetics | 0.864 | 0.0315 | 0.817-0.903 |  | 0.884 | 0.0367 | 0.817-0.932 |  | 0.6792 |
| Receiving DRT 5 years |  |  |  |  |  |  |  |  |  |
| Clinical | 0.710 | 0.0361 | 0.645-0.770 |  | 0.687 | 0.0621 | 0.585-0.777 |  | 0.7488 |
| Clinical-Genetics | 0.798 | 0.0316 | 0.738-0.850 |  | 0.879 | 0.0390 | 0.798-0.936 |  | 0.1066 |

AUC, area under the curve; SE, standard error; 95%CI, 95% confidence intervals; DRT, dopamine replacement therapy

**Table S6 Comparison of different prediction models between the derivation group and subgroups without special PD causative genes mutation**

| Models | Derivation group | | |  | Group 1 | | |  | *P*-value ^a^ | Group 2 | | |  | *P-*valu*e ^b^* |
| --- | --- | --- | --- | --- | --- | --- | --- | --- | --- | --- | --- | --- | --- | --- |
|  | AUC | SE | 95%CI |  | AUC | SE | 95%CI |  |  | AUC | SE | 95%CI |  |  |
| Duration of first 5 years* |  |  |  |  |  |  |  |  |  |  |  |  |  |  |
| Clinical | 0.714 | 0.0461 | 0.655-0.767 |  | 0.716 | 0.0454 | 0.658-0.770 |  | 0.9965 | 0.713 | 0.0459 | 0.652-0.769 |  | 0.9877 |
| Clinical-Genetics | 0.864 | 0.0315 | 0.817-0.903 |  | 0.862 | 0.0319 | 0.815-0.901 |  | 0.9644 | 0.883 | 0.0288 | 0.826-0.920 |  | 0.6562 |
| Receiving DRT 5 years^#^ |  |  |  |  |  |  |  |  |  |  |  |  |  |  |
| Clinical | 0.710 | 0.0361 | 0.645-0.770 |  | 0.723 | 0.0360 | 0.658-0.781 |  | 0.7987 | 0.729 | 0.0379 | 0.662-0.788 |  | 0.7166 |
| Clinical-Genetics | 0.798 | 0.0316 | 0.738-0.850 |  | 0.795 | 0.0319 | 0.736-0.847 |  | 0.9467 | 0.812 | 0.0326 | 0.752-0.863 |  | 0.7578 |

*Duration of first 5 years, Derivation group (n=279); Group 1 (n=267), 12 patients with mutation in *parkin*, *PINK1* and *DJ1* were excluded; Group 2 (n=257), 22 patients with mutation in *SNCA*, *LRRK2*, *parkin*, *PINK1*, *DJ1*, *PLA2G6*, *VPS35* were excluded.

^#^ Receiving DRT 5 years, Derivation group (n=232); Group 1 (n=222), 10 patients with mutation in *parkin*, *PINK1* and *DJ1* mutation were excluded; Group 2 (n=214), 18 patients with mutation in *SNCA*, *LRRK2*, *parkin*, *PINK1*, *DJ1*, *PLA2G6*, *VPS35* were excluded.

^a^ Comparison between Derivation group and Group 1; ^b^ Comparison between Derivation group and Group 2

**Table S7 Single-factor association with LIDs incidence in the multivariable non-linear models**

| Factors | Multivariable *P* value | OR [95%CI] |
| --- | --- | --- |
| First 5 years (n=279)* |  |  |
| LEDD | **3.4e-4** |  |
| Duration | 0.011^&^ |  |
| *DRD3* rs6280: AG genotype | 0.173 | 0.57[0.26-1.28] |
| *DRD3* rs6280: GG genotype | **0.001** | 7.20[2.14-24.15] |
| *SLC6A3* rs460000: GT genotype | 0.015^&^ | 4.58[1.35-15.59] |
| *SLC6A3* rs460000: TT genotype | 0.023^&^ | 4.58[1.24-16.94] |
| *HRAS* rs12628: CT genotype | **0.005** | 0.22[0.08-0.63] |
| *BDNF* rs6265: CT genotype | 0.149 | 0.54[0.23-1.25] |
| *BDNF* rs6265: TT genotype | 0.050^&^ | 0.32[0.11-0.92] |
| Receiving DRT for 5 years (n=232)^#^ |  |  |
| LEDD | **1.7e-4** | - |
| *DRD3* rs6280: AG genotype | 0.668 | 0.86[0.44-1.68] |
| *DRD3* rs6280: GG genotype | 0.010^&^ | 4.20[1.41-12.58] |
| *GRIN2B* rs1860201: CT genotype | 0.873 | 1.07[0.47-2.41] |
| *GRIN2B* rs1860201: TT genotype | 0.048^&^ | 2.37[1.00-5.60] |
| *COMT* rs4680: AG genotype | 0.986 | 0.99[0.51-1.94] |
| *COMT* rs4680: AA genotype | 0.034^&^ | 3.84[1.10-13.36] |
| *HRAS* rs12628: CT genotype | 0.029^&^ | 0.43[0.20-0.92] |

Variables whose p value less than 0.15 from logistic regression were put into stepwise regression; *including duration, initial LEDD (levodopa equivalent daily dose), *DRD3* rs6280, *DRD2* rs1800497, *SLC6A3* rs460000, *HRAS* rs12628, *COMT* rs4680 and *BDNF* rs6265; ^#^ including initial symptoms, family history, initial LEDD (levodopa equivalent daily dose), *DRD3* rs6280, *SLC6A3* rs460000, *GRIN2B* rs1806201, *HRAS* rs12628, *COMT* rs4680 and *TOR1A* rs1801968; ^&^no significance after Bonferroni correction.


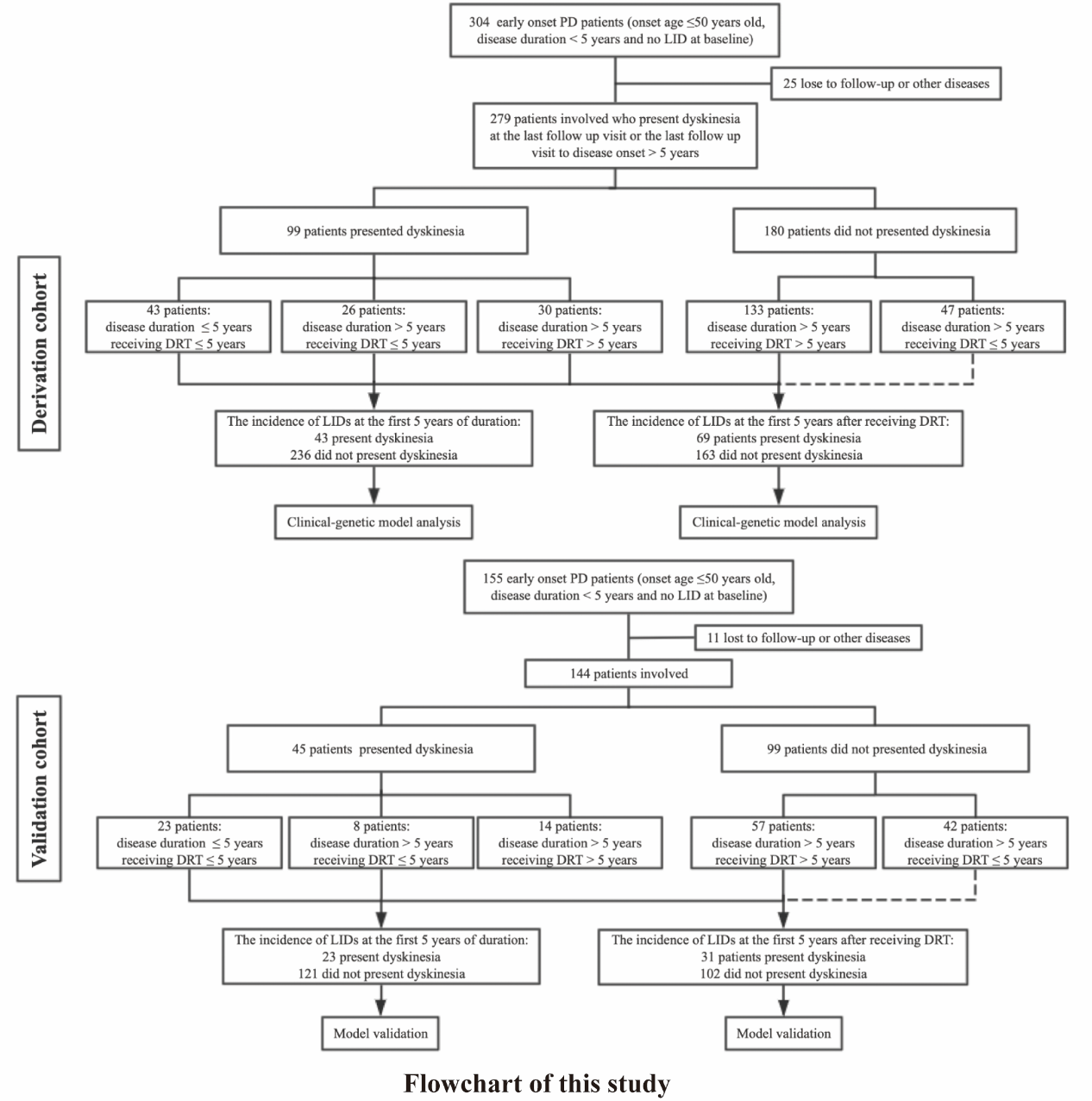


Fig. S1. Flowchart of the study. The dotted lines represent patients not included in the analysis of LID incidence during the first 5 years of DRT.
